# Overexpression of a Chimeric Gene, *OsDST-SRDX,* Improved Salt Tolerance of Perennial Ryegrass

**DOI:** 10.1038/srep27320

**Published:** 2016-06-02

**Authors:** Huifang Cen, Wenxing Ye, Yanrong Liu, Dayong Li, Kexin Wang, Wanjun Zhang

**Affiliations:** 1 Department of Grassland Science, China Agricultural University, Beijing, 100193, P. R. China; 2 State Key Laboratory of Plant Genomics and National Center for Plant Gene Research, Institute of Genetics and Developmental Biology, Chinese Academy of Sciences, Beijing 100101, P. R. China; 3 National Energy R&D Center for Biomass (NECB), China Agricultural University, Beijing, 100193, P. R. China

## Abstract

The Drought and Salt Tolerance gene (*DST*) encodes a C_2_H_2_ zinc finger transcription factor, which negatively regulates salt tolerance in rice (*Oryza sativa*). Phylogenetic analysis of six homologues of *DST* genes in different plant species revealed that *DST* genes were conserved evolutionarily. Here, the rice *DST* gene was linked to an SRDX domain for gene expression repression based on the Chimeric REpressor gene-Silencing Technology (CRES-T) to make a chimeric gene (*OsDST-SRDX*) construct and introduced into perennial ryegrass by *Agrobacterium*-mediated transformation. Integration and expression of the *OsDST-SRDX* in transgenic plants were tested by PCR and RT-PCR, respectively. Transgenic lines overexpressing the *OsDST-SRDX* fusion gene showed obvious phenotypic differences and clear resistance to salt-shock and to continuous salt stresses compared to non-transgenic plants. Physiological analyses including relative leaf water content, electrolyte leakage, proline content, malondialdehyde (MDA) content, H_2_O_2_ content and sodium and potassium accumulation indicated that the *OsDST-SRDX* fusion gene enhanced salt tolerance in transgenic perennial ryegrass by altering a wide range of physiological responses. To our best knowledge this study is the first report of utilizing Chimeric Repressor gene-Silencing Technology (CRES-T) in turfgrass and forage species for salt-tolerance improvement.

The area of saline land worldwide is nearly 1 billion hectares, and accounts for 10 percent of the total land area^1^,^2^. Salinity stress has become one of the major abiotic factors that severely affects plant growth. Perennial ryegrass (*Lolium perenne* L.) is an important cool-season grass in temperate regions worldwide. It is widely cultivated as a turfgrass and forage with favorable agronomic traits, including rapid establishment rate, strong tiller ability, strong trample resistance, as well as high yield^3^. However, the growth of perennial ryegrass as turfgrass is hampered by the aggravation of soil salinization and the shortage of water resources. Therefore, it is necessary to improve the salt tolerance of perennial ryegrass. However, perennial ryegrass is a cross-pollinated, self-infertile plant, resulting in slow progress in breeding new varieties with conventional strategies. In recent years, genetic engineering has been widely used in plant genetic improvement and has showed obvious advantages. Breeding new varieties of perennial ryegrass with enhanced salt tolerance through genetic engineering is expected^3^,^4^.

The application of biotechnology to ryegrass was initiated early. In 1977, fertile perennial ryegrass regeneration plants were obtained by using the shoot-tip meristem as an explant^5^. Later, mature embryos, immature inflorescence, leaf, and meristem cells of perennial ryegrass were used as explants for perennial ryegrass regeneration^6^,^7^,^8^,^9^,^10^. Transgenic ryegrass plants were first obtained in 1999 by using silicon carbide fiber-mediated transformation^11^. In 2005, *Agrobacterium*-mediated transformation of perennial ryegrass was reported^12^. Thereafter, high-efficiency *Agrobacterium*-mediated transformation systems of perennial ryegrass were established^13^,^14^,^15^. Particularly, transformation efficiency of perennial ryegrass was improved extensively by reducing defense responses of calluses in *Agrobacterium*-mediated transformation, which made a solid foundation for functional-gene research and genetic improvement of perennial ryegrass^15^.

In recent years, a growing number of reports focused on model plants and crops have indicated that salt tolerance could be improved by genetic transformation^4^,^16^,^17^. Currently, tens of different salt-resistant plant species have been obtained by genetic transformation. And nearly 40 transcription factors have reportedly been associated with plant salt resistance^18^, such as MYB^16^,^19^, NAC^20^,^21^, bZip^22^ and DREB^23^,^24^ transcription factors. Overexpression of genes related to the Na^+^/H^+^ antiporter also significantly improved the salt resistance of transgenic plants. For instance, overexpression of a rice vacuolar Na^+^/H^+^ antiporter gene, *OsNHX1,* in perennial ryegrass significantly increased the salt resistance of transgenic plants^12^. Overexpression of an *AVP1* gene of *Arabidopsis* in creeping bentgrass significantly increased the salt tolerance of transgenic plants^25^.

In addition, some transcriptional factors negatively regulate the stress resistance of plants. Drought and Salt Tolerance (DST) is a zinc finger transcription factor which is negatively related to drought and salt tolerance of plants^26^. *DST* regulates signal transduction pathways of stomatal closure induced by H_2_O_2_, and directly modulates genes related to H_2_O_2_ homeostasis to regulate stomatal closure. The rice *DST* mutant (*dst*) lacks the protein-coding function and results in reduced stomatal density, consequently enhancing drought and salt tolerance in rice. *DST* has also been revealed to directly regulate the expression of *OsCKX2* in the apical meristem, and by improving the cytokinin content of the apical meristem to improve the activity of the meristem, thereby increasing rice tiller numbers, eventually improving rice yield^27^. Recently, Cui *et al.*^28^ demonstrated a co-activator of DST (DCA1), which interacted with DST to regulate the expression of genes related to H_2_O_2_ homeostasis, such as *peroxidase 24 precursor (Prx 24)*, to negatively regulate stomatal closure. The *DCA1-DST-Prx24* pathway contributed to drought and salt tolerance in rice.

Chimeric REpressor gene-Silencing Technology (CRES-T) was developed as a specific technology for gene silencing, mainly used for analyzing the function of plant transcription factors^29^,^30^. By linking a SRDX-motif to the C-terminal of transcription activators, the chimeric gene has been changed into highly efficient negative regulons to repress the expression of target genes specifically and efficiently^31^,^32^. In recent years, this technology has been widely used for analyzing plant functional genes of transcription factors. The repression domain SRDX fused with *AtMBF1c* significantly reduced the germination rate of transgenic *Arabidopsis* and also made plants dwarf^33^. The SRDX domain fused with *SlER24* prolonged the germination time of transgenic tomato and also affected plant growth^33^. The fusion gene *ARR1-SRDX* enhanced the resistance of transgenic plants to cytokinin, and decreased the cytokinin content, finally resulting in small leaves, large roots and seeds^34^.

In this report, a SRDX-motif was linked to the C-terminal of the rice zinc finger DST to make a chimeric gene *OsDST-SRDX*. Then the *OsDST-SRDX* chimeric gene was introduced into the perennial ryegrass genome by *Agrobacterium*-mediated transformation to produce a new genotype of perennial ryegrass with enhanced performance under salinity conditions. Plant phenotypes, growth and physiological responses were studied carefully in both transgenic and non-transgenic plants under different salt stress conditions. The results indicated that *OsDST-SRDX* enhanced salt tolerance of transgenic perennial ryegrass remarkably.

## Results

### The *DST* gene exists in perennial ryegrass and has responses to salt stress

**Figure 1:**
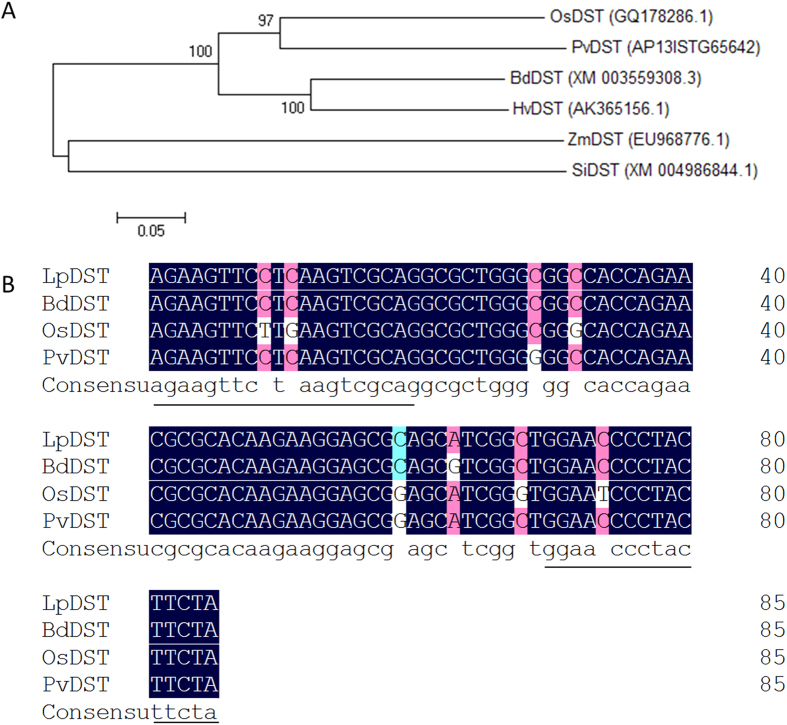
Phylogenetic analysis of six DSTs and the conserved nucleotide sequence alignment of four *DSTs.* (**A**) phylogenetic analysis of six DSTs derived from different plant species (*Hordeum vulgare*, *Panicum virgatum*, *Oryza sativa*, *Brachypodium distachyon*, *Setaria italic* and *Zea mays*), the Neighbor-Joining phylogenetic tree was constructed using MEGA 5.1; (**B**) the nucleotide sequence alignment between the conserved sequence fragment of *OsDST, PvDST, BdDST* and *LpDST*, underlined was the pair of primers used to amplify the conserved domain of *LpDST* in perennial ryegrass.

A Neighbor-Joining phylogenetic tree of six DSTs derived from different plant species (*Hordeum vulgare*, *Panicum virgatum*, *Oryza sativa*, *Brachypodium distachyon*, *Setaria italic* and *Zea mays*) was constructed, which demonstrated that the *DST* gene was conserved evolutionarily in C3 and C4 plants (Fig. 1A). Through nucleotide sequence alignment, we found three *DSTs* (*OsDST*, *BdDST* and *PvDST*) showed high similarity (Fig. S1). According to the conserved nucleotide sequence in alignment between *OsDST*, *BdDST*, *PvDST*, *HvDST*, *SiDST* and *ZmDST*, a pair of primers was designed, using Primer Premier 5.0, to amplify a conserved sequence fragment (85 bp) of the *LpDST* gene from the cDNA derived from leaves of perennial ryegrass (Fig. S1). By sequence alignment, a high-sequence identity (97.35%) was found between the sequence of the cloned perennial ryegrass fragment (*LpDST*) and the conserved sequence of *BdDST*, *OsDST* and *PvDST*; the pair of primers for *LpDST* gene fragment amplification was marked with underlining (Fig. 1B). The sequence identity between the conserved nucleotide fragments between the *DSTs* indicated that the *DST* gene existed in the genome of perennial ryegrass and might be related to the drought and salt tolerance of perennial ryegrass.

**Figure 2:**
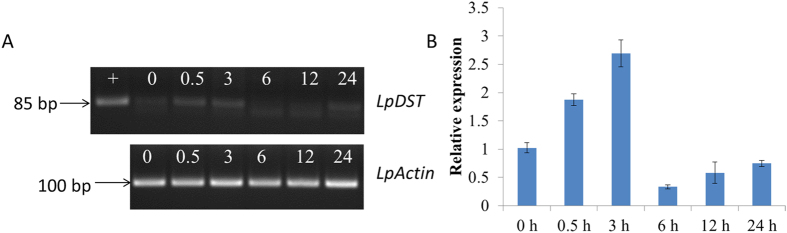
Relative expression of *LpDST* under 300 mM NaCl treatment. (**A**) semi-quantitative RT-PCR analysis of the relative expression of the endogenous *DST* gene under different salinity treatment time; the perennial ryegrass internal gene *LpActin* was used as a template loading control, +, positive control, the *OsDST-SRDX* plasmid as a template; 0–24 h, different sampling time points; (**B**) real-time quantitative PCR tests of the relative expression of the endogenous *LpDST* gene under different salinity treatment times. Error bars represent means ± SE (n = 3).

To identify whether the *DST* gene expression was related to salt stress or not, a semi-quantitative RT-PCR test was carried out with the pair of primers for *LpDST* gene fragment amplification, cDNAs derived from leaves of the wild type (WT) perennial ryegrass after 0 h, 0.5 h, 3 h, 6 h, 12 h and 24 h salinity treatment respectively under 300 mM NaCl as the template. The results showed that the expression levels of the endogenous *LpDST* gene in perennial ryegrass was up-regulated after 0.5 h and 3 h of salinity treatment, then was down-regulated and remained at a low level with the prolonging of the salinity treatment time (Fig. 2A). The internal reference gene *LpActin* of perennial ryegrass served as a template loading control.

To further verify the results of RT-PCR, a real-time quantitative PCR was applied with the same primers and template. The results showed that the relative expression levels of the endogenous *DST* gene of perennial ryegrass were increased nearly 1-fold and 2-fold respectively after 0.5 h and 3 h of salinity treatment. And then was rapidly down-regulated after 6 h of salinity treatment and remained at a relatively stable and low expression level (Fig. 2B). The results indicated that the endogenous *DST* gene may play an important role in perennial ryegrass response to salt stress, and lower expression of the *DST* gene is favorable to salt tolerance of perennial ryegrass.

### Production and verification of *OsDST-SRDX* transgenic perennial ryegrass

By following the method reported by Zhang *et al.*^15^, embryonic callus derived from mature seeds of perennial ryegrass (cultivar: Citation IV) were transformed by *Agrobacterium.* The *Agrobacterium* strain harboring a plasmid pZH01_*OsDST-SRDX* (T-DNA region is showed in Fig. S2A) was used for transformation. To obtain resistant callus, the calluses were screened with 50 and 100 mg L^−1^ *hpt* after co-cultivation. Resistant regeneration shoots were obtained on differentiation medium with 50 mg L^−1^ *hpt* (Fig. S2B). After rooting culture (Fig. S2C), the resistant regenerated plantlets were transplanted to pots (Fig. S2D).

**Figure 3:**
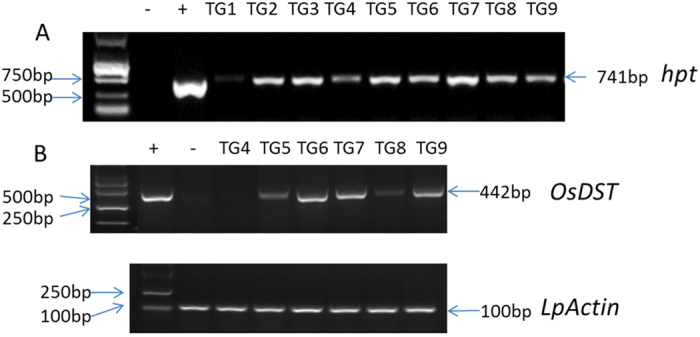
Verification of the *OsDST-SRDX* transgenic perennial ryegrass plants. (**A**) PCR tests of the selectable marker gene *hpt;* +, positive control, DNA of plasmid pZH01*_OsDST-SRDX* was used as a template; −, negative control, the DNA of the WT plants was used as a template; TG1-TG9 shows the DNA of transgenic plants was used as a template; (**B**) semi-quantitative RT-PCR analysis of the relative expression of the *DST* gene in the transgenic lines of TG4-TG9; the perennial ryegrass internal gene *LpActin* was used as a template loading control.

The resistant transgenic plants were subjected to PCR tests with a pair of primers specific to the selectable marker gene *hpt*. As shown in Fig. 3A, the expected size of the DNA fragment (741 bp) was detectable in all of the tested transgenic (TG) plants, while WT plants had no such an amplification product. The results preliminarily confirmed that the exogenous gene *OsDST-SRDX* has integrated into the genome of resistant perennial ryegrass.

For further analysis, PCR-positive plants were selected randomly for reverse transcriptional PCR to test the expression of transgene, *OsDST*, in the transcriptional level. The expected size of the *OsDST* gene fragment (442 bp) was amplified in transgenic lines of TG5 to TG9 by using a pair of *OsDST* gene-specific primers (Fig. 3B), which demonstrated that *OsDST-SRDX* in the transgenic plants was expressed in the transcriptional level. However, the target fragment was not showed in the transgenic line TG4, which might result from the transgene having not been expressed. Compared with the amplification result of a perennial ryegrass internal reference gene *LpActin* (Fig. 3B) which served as a template loading control, the result indicated that transgenic lines TG6, TG7 and TG9 exhibited relatively higher expression levels of the *DST* gene.

### *OsDST-SRDX* gene altered phenotypes of transgenic perennial ryegrass

**Figure 4:**
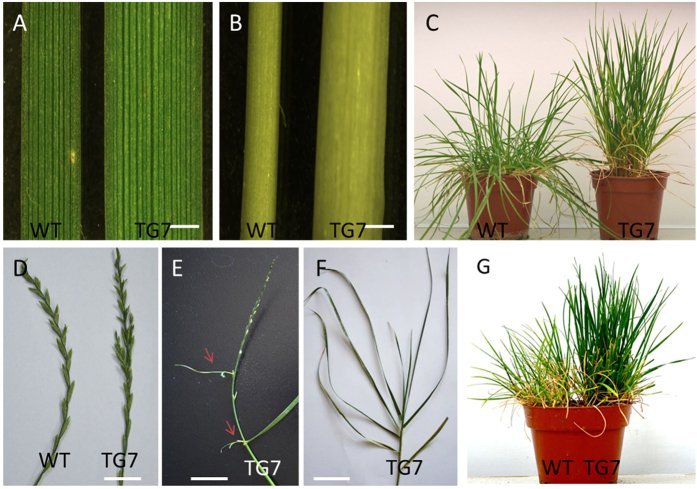
Morphological comparisons of the WT and TG plants. (**A**) fully expanded leaves of the WT and TG plants under an anatomical lens, scale bar, 1 mm; (**B**) stems image of the WT and TG plants under anatomical lens, scale bar, 1 mm; (**C**) phenotypes of three-month-old WT and TG plants initiated from the same amount of tillers; (**D**) inflorescence phenotype of the TG and WT plants, scale bar, 1 cm; (**E**) inflorescence phenotype of the TG plants, the arrows point to new buds, scale bar, 1 cm; (**F**) phenotype of a single tiller of the TG plants at the jointing stage under low-light condition, scale bar, 1 cm; (**G**) phenotype of the TG and WT plants after a salt shock treatment with 300 mM NaCl for one week, the TG plants stayed green, while the WT plant was obviously wilted.

The *OsDST-SRDX* transgenic plants showed obvious different phenotypes compared with the wild-type perennial ryegrass. Under the same conditions, the TG plants showed obvious growth advantages, with higher plant height and broader leaf width and stem diameter than the WT plants. The transgenic lines showed compacted, erect growth, which is distinct from the WT plants, obviously. Compared with the WT plants, the blade width of the TG plants was much wider (Fig. 4A). Seven months after being transplanted in a green house, the blade width of the TG5 was 3.33 mm, the TG7 was 3.42 mm, which were all remarkably wider than the 2.20 mm and 2.74 mm of the WT plants that were regenerated from tissue culture, both TG and WT plants were fully developed (Table 1). The stems of the TG plants were also significantly thicker than those of WT plants (Fig. 4B), but there were no significant differences among transgenic lines (Table 1). And the vertical heights of the TG plants were significantly higher than those of the WT plants (Fig. 4C). Besides that, the inflorescence of the TG and WT plants was also discriminating: several lateral buds appeared on the inflorescence of the TG plants at the full-bloom stage (Fig. 4D,E). Under low-light conditions, the tillers of the TG plant have more leaves than do the tillers of the WT plants at the jointing stage (Fig. 4F).

### Overexpression of *OsDST-SRDX* fusion gene enhanced salt tolerance of transgenic perennial ryegrass

To test whether the salt tolerance of transgenic perennial ryegrass was improved by overexpressing the *OsDST-SRDX* fusion gene, we watered transgenic lines with 300 mM NaCl solution directly to test their responses to salt shock. As showed in Fig. 4G, transgenic lines were more resistant to salt shock and showed no obvious salt injury symptoms after watering with NaCl solutions for a week. On the contrary, WT plants exhibited obvious salt injury symptoms, with leaves showing obvious chlorosis and wilting. The transgenic line TG7 showed the best performance and was chosen for detailed salt-tolerance tests.

**Figure 5:**
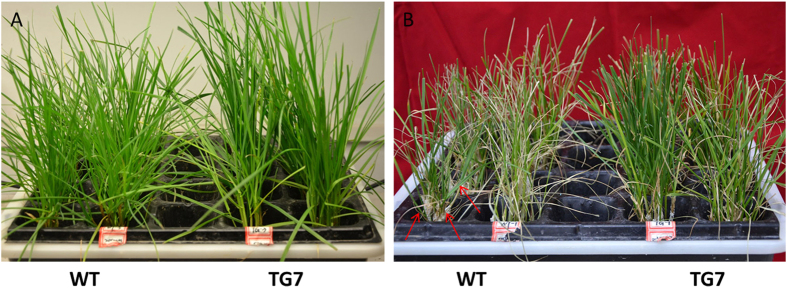
Responses of TG and WT plants to 300 mM of NaCl treatment before (**A**) and after (**B**) 21 days. The arrows point out NaCl precipitation at the base of the stem.

Before salinity treatment, replicates divided from tillers of the TG and WT plants after cultivating in 1/2× Hoagland solution were trimmed to a uniform level to achieve uniform growth both on the above ground part and the roots. Afterwards, they were randomly planted in 1/2× Hoagland solution supplemented with various concentrations of NaCl (0 mM, 100 mM, 200 mM and 300 mM). Three days later, salt precipitation on the base of the stem was visible, and some leaves of both the TG and WT plants began to wilt. The phenomena of hindered shoot and root development became more severe with the increas of salt concentration. In addition, the WT plants exhibited more severe growth inhibition and tissue damage than TG plants under the same salinity treatment condition. After 4 days of treatment, curled and wilted leaves in the treatments of NaCl concentrations over 200 mM were all found both on the TG and WT plants. Overall, the WT plants exhibited more severely wilted leaves and poorer growth than the TG plants. After 21 days of salinity treatment, the TG plants obviously performed better than WT plants under all the tested salt concentrations. Under 300 mM of NaCl treatment, the WT plants all died, whereas the TG plants still survived and showed slight growth with some green leaves (Figs 5 and S3).

### The *OsDST-SRDX* transgenic plants showed higher water retention capacity, less cell membrane damage, lower lipid oxidative level and lower proline content than the WT plants under salt stress

**Figure 6:**
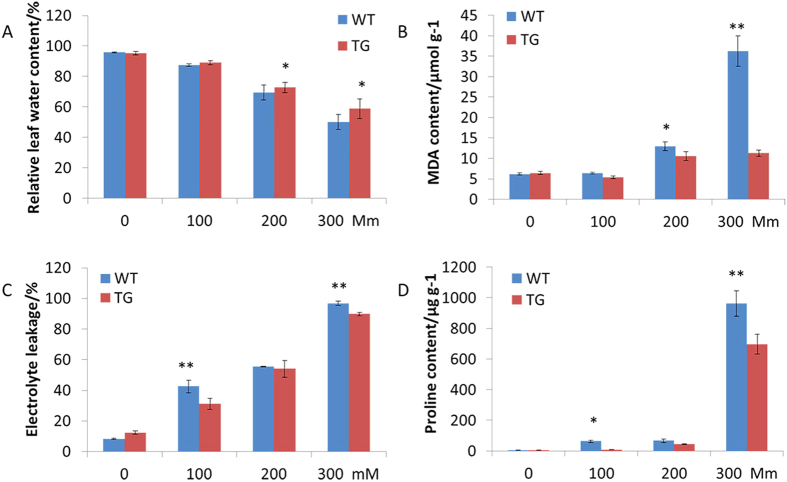
The effect of various concentrations of NaCl (0, 100, 200 or 300 mM) treatment on the RWC (**A**), the MDA content (**B**), the EL (**C**), and the proline content (**D**) 12 days after treatments. Data are presented as means of three technical replicates, and error bars represent means ± SE. Asterisks indicate a significant difference of RWC, electrolyte leakage, MDA content and proline content between TG and WT plants at *p* < 0.05 or 0.01 by Student’s t-test.

**Figure 7:**
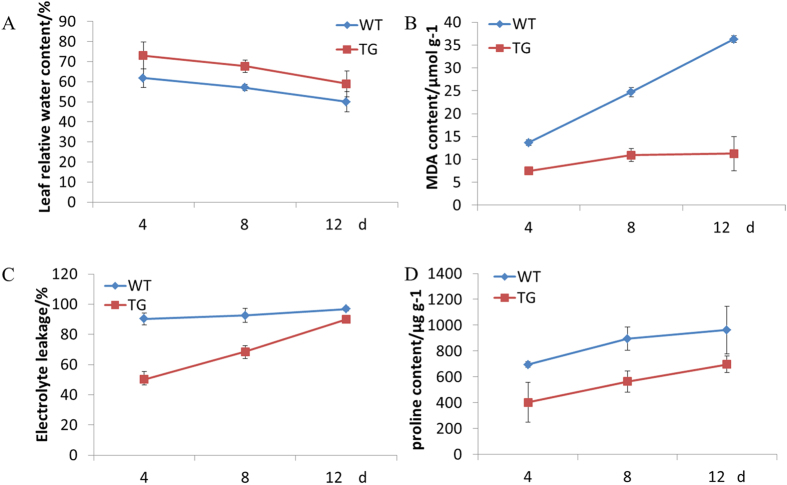
The variation tendency of the RWC (**A**), the MDA content (**B**), the EL (**C**) and the proline content (**D**) under 300 mM of NaCl treatment at 4, 8 or 12 d. Data are presented as means of three technical replicates, and error bars represent means ± SE.

Relative water content (RWC) was measured to assess the leaf water status of the TG and WT plants. No significant difference was observed between the TG and WT plants in the NaCl-free condition. However, when exposed to various concentrations of NaCl, significant differences in RWC were observed between the TG and WT plants. Under salinity conditions and with the increase of the NaCl concentration, the RWC of both the TG and WT plants declined, but this decline was more significant in the WT plants after 12 days of salt treatment. The RWC of TG plants was always higher than that of the WT plants (Fig. 6A). And with the prolonged salinity treatment with 300 mM of NaCl, the RWC of both TG and WT plants declined. After twelve days of treatment, the RWC of the WT plants was reduced to 49.9%, which was significantly lower than that of the TG plants, 58.9% (Fig. 7A). The results indicated that the *OsDST-SRDX* transgenic plants had greater water-retention capacity than the WT plants.

MDA is one of the final products of membrane lipid peroxidation under stresses^35^. MDA content reflects the level of membrane lipid peroxidation. In this study, MDA content of both the TG and WT plants was measured to investigate the levels of the plants membrane lipid peroxidation due to salt stress. No significant difference was observed between the TG and WT plants under NaCl-free condition, MDA content was about 6.43 μmol g^−1^. However, significant differences of MDA content were observed between the TG and WT plants under various concentrations of NaCl treatments (100 to 300 mM) for 12 days. And with the increasing of NaCl concentration, MDA content of both the TG and WT plants was raised. Under 300 mM of NaCl condition, with the increase of salinity treatment time, the MDA content of the WT plants increased sharply, but that of the TG plants increased more gently (Fig. 7B). After 12 days at 300 mM NaCl treatment, the MDA content of the WT plants had reached to 36.25 μmol g^−1^, while that of the TG plants was only 11.28 μmol g^−1^ (Fig. 6B). The results demonstrated that the *OsDST-SRDX* fusion gene reduced the degree of membrane lipid oxidation of the transgenic plants under salt stresses.

When plants were exposed to abiotic stresses, their cell membrane was damaged. And membrane permeability increased, finally resulting in electrolyte leaking from cells. To investigate the degree of cell membrane damage under salt stress, we measured the electrolyte leakage (EL) of both the TG and WT plants under normal and salinity conditions. Under normal conditions, no significant difference of the EL was observed between the TG and WT plants. The EL remained at 10~12%. However, significant differences were observed between the TG and WT plants when various concentrations of NaCl were applied. After 12 days, with the increase of NaCl concentration, the EL of both the TG and WT plants was increased, but the EL of the WT plants was considerably higher than that of the TG plants under the same salinity conditions (Fig. 6C). Four days after salinity treatment with 300 mM NaCl, the EL of the WT plants increased to 90.2%, indicating the leaf cell membrane had been severely damaged, while that of the TG plants was only 50.3%. After 12 days under 300 mM of NaCl, the EL of the WT plants was about 96.8%, and that of the TG plants was 89.9% (Fig. 7C). The results indicated that the *OsDST-SRDX* protected the membrane of the TG plants, hence, enhanced the salt tolerance.

Proline is one of the primary osmotic regulation substances in plant cells under salt stress. Most plants showed increased proline content under stress conditions, which was regarded as correlated to their stress resistance. In this study, the proline content of both the TG and WT plants was tested under different salinity conditions. The proline content of both the TG and WT plants was at a low level at the NaCl-free condition, and no significant difference of proline content was observed. However, when exposed to various concentrations of NaCl, significant differences of proline content of both the TG and WT plants were detected. But the proline content of the WT plants increased more rapidly with the increase of the NaCl concentration. In the same concentration of NaCl condition, the proline content of the TG plants was always lower than that of the WT plants. After 12 days under 100 mM of NaCl, the proline content of WT plants was 63.0 μg g^−1^, significantly higher than that 7.76 μg g^−1^ of the TG plants. After 12 days under 300 mM of NaCl, proline content of WT plants was 961.57 μg g^−1^, while that of TG plants was 571.60 μg g^−1^ (Fig. 6D). And also, with the increase of salinity treatment time under 300 mM of NaCl, the proline content of both the TG and WT plants rose, but the proline content of the TG plants was significantly lower than that of the WT plants (Fig. 7D), which indicated that the TG plants were less stressed than the WT plants.

### The *OsDST-SRDX* transgenic plants accumulated less Na^+^ and more K^+^

**Figure 8:**
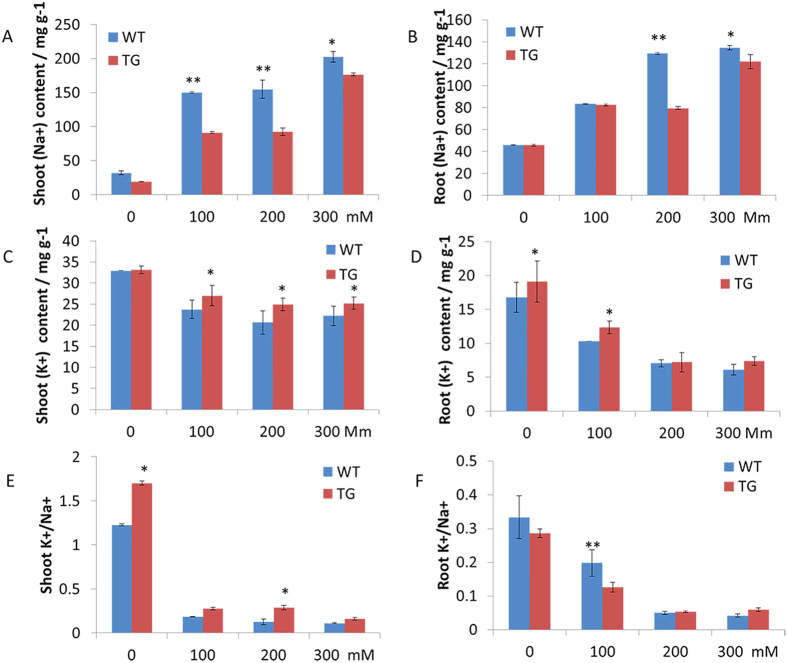
Mineral content of the TG and WT plants under normal and various concentrations of NaCl (100, 200 and 300 mM) (**A**) Na^+^ content in shoot; (**B**) Na^+^ content in root; (**C**) K^+^ content in shoot; (**D**) K^+^ content in root; (**E**) K^+^/Na^+^ in shoot; (**F**) K^+^/Na^+^ in root. Data are presented as means of three technical replicates, and error bars represent means ± SE. Asterisks indicate a significant difference of Na^+^, K^+^, K^+^/Na^+^ in shoot and root between the TG and WT plants at *p* < 0.05 or 0.01 by Student’s t-test.

To detect how the *OsDST-SRDX* fusion gene affected Na^+^ and K^+^ uptake in the transgenic plants, we measured shoot and root Na^+^ and K^+^ contents in the TG and WT plants after they were treated with different concentrations of NaCl (0, 100, 200 and 300 mM) for 12 days, respectively. No noteworthy differences of Na^+^ content in either shoot or root were observed between TG and WT plants under normal conditions. However, when exposed to various concentrations of NaCl (100, 200 and 300 mM), significant differences were observed between the TG and WT plants except root Na^+^ content under 100 mM of NaCl concentration. And with the increase of NaCl concentrations, Na^+^ accumulation in the shoots and roots of both the TG and WT plants was increased, but the uptake of Na^+^ level in the shoots and roots of the TG plants was always significantly lower than that of the WT plants in the same concentration of NaCl (Fig. 8A,B). Under normal conditions, K^+^ levels in the shoots of the TG and WT plants were similar. When NaCl concentration increased, shoot K^+^ levels declined in both the TG and WT plants with the TG plants having significantly higher K^+^ (Fig. 8C). When the plants were subjected to a lower concentration of NaCl (100 mM), a dramatic decrease in root K^+^ content was observed in the TG and WT plants. However, no difference of root K^+^ level was observed between the TG and WT plants when exposed to higher concentrations of NaCl (200 and 300 mM) (Fig. 8D). The results suggested that perennial ryegrass plants might have the ability to maintain K^+^ homeostasis under higher salinity stresses. For the K^+^/Na^+^ ratio, a dramaticly higher K^+^/Na^+^ ratio was observed in the shoots of the TG plants than those of the WT plants in both 0 mM and 200 mM NaCl treatments, whereas no significant differences were observed in the treatments with 100 mM and 300 mM NaCl (Fig. 8E). However, under normal and lower salinity conditions, the K^+^/Na^+^ ratio in roots of the TG plants was lower than that of the WT plants. The K^+^/Na^+^ ratio in the roots of TG plants was slightly higher than that of WT plants in 200 and 300 mM NaCl (Fig. 8F). The results indicated that the *OsDST-SRDX* fusion gene is beneficial to keeping a higher K^+^/Na^+^ ratio in the shoots of transgenic plants.

## Discussion

Genetic engineering is an alternative method to breed new varieties with high salt tolerance. Many genes and techniques have been used to enhance plant salt tolerance^12^,^25^,^36^,^37^. Chimeric REpressor gene-Silencing Technology (CRES-T) is an effective technology of gene silencing, following antisense RNA and RNAi technology, which has been established on the basis of the EAR type transcription repressor^29^,^32^. By linking the SRDX-motifs to the C-terminus of transcription activators, the transcriptional factors were changed into highly efficient negative regulons to repress the expression of target genes^30^. This technique has been used for analyzing the function of plant transcription factors and modifying interesting traits of plants through genetic transformation^31^,^38^,^39^. In recent years, CRES-T has been successfully used in *Arabidopsis*^40^, cyclamen flowers^41^, pharbitis nil^42^ and rice^43^. Utilization of CRES-T on forage and turfgrass has not been reported yet. In this study, a chimeric gene, *OsDST-SRDX,* was introduced into the genome of perennial ryegrass, with the expectation of producing transgenic perennial ryegrass with an inhibited transcriptional activation function of endogenous *LpDST* and thus to enhance the salt tolerance of perennial ryegrass. The results demonstrated that the salt tolerance of perennial ryegrass was improved by overexpressing the *OsDST-SRDX* fusion gene, which indicated that the *DST* regulation pathway also exists in perennial ryegrass and that the CRES-T technique could be used to modify the salt tolerance trait of perennial ryegrass. In addition, the result of phylogenetic analysis indicated that *DST* genes were highly conserved in different plant species, which means that there is a great potential for the *DST-SRDX* strategy to be widely applied in the salt-tolerance improvement of other agriculturally important crop species.

Compared to CRES-T, microRNA-based genetic modification technology is also a strategy used for plant trait modification. However, some potential risks of miRNA-based GM technology also exist in crop genetic modification, such as off-target, transgene introgression and gene silencing^44^. In this report, CRES-T was used for perennial ryegrass improvement, and the transgenic plants showed enhanced salinity tolerance. CRES-T may provide a smart strategy to solve the potential problems in microRNA-based technologies.

In this report according to the *Agrobacterium*-mediated genetic transformation system of perennial ryegrass established by Zhang *et al.*^15^, transgenic perennial ryegrass overexpressing the *OsDST-SRDX* chimeric gene was produced. Transgenic lines exhibited upright growth, higher plant height, thicker stems and wider leaves than the WT plants, which were consistent with the obvious wider leaf width, the large number of panicles and the longer main panicle length of the reported rice *dst* mutants^26^. *DST* regulates signal transduction pathway of stomatal closure induced by H_2_O_2_ and directly modulates the gene expression related to H_2_O_2_ homeostasis and hence negatively regulates stomatal closure^26^. The protein-coding function of *DST* is lost in the *dst* mutant and resulted in reduced stomatal density, consequently resulting in enhanced drought and salt tolerance in rice. A recent study in creeping bentgrass showed that overexpressing *Osa-miR319* exhibited improved salt and drought tolerance in transgenic plants also with remarkably wider leaves and thicker stems^45^,^46^, but the detailed mechanism still needs to be elucidated. The *OsDST-SRDX* transgenic perennial ryegrass plants showed erect plant growth and compact tillers, which not be desirable traits for turfgrass but might be good for forage production. The strong stems and upright growth characteristics of the *OsDST-SRDX* transgenic plants might also be favorable for plant lodging resistance, because the lodging resistance plants normally have the characteristics of strong stems and upright growth^47^. In this report, we also revealed that the *DST* transcription factor was linked to salt tolerance in perennial ryegrass, but the regulation mechanism calls for further research.

MDA is one of the products of membrane lipid peroxidation and cytotoxicity, which can combine and cross-link with proteins and enzymes on the membrane to make it inactive and consequently to destruct membrane structure^35^. MDA content is usually used to reflect the damage extent of plant cells under stress conditions. In our study, the MDA content of both the TG and WT plants was improved with the increase of salinity treatment strengths, but the MDA content of the TG plants was always lower than that of the WT plants, which demonstrated that the extent of the membrane lipid peroxidation of the TG plants is much lower, and the TG plants showed strong tolerance to salt stress. Electrolyte leakage and relative water content tests in our study also showed that the TG plants displayed improved resistance than that of the WT plants against salt stresses.

Previous research has demonstrated that adversity stresses such as drought and salinity can cause osmotic responses in plants, and some organic osmolytes, such as proline or glycine betaine could accumulate to decrease the water potential in plants to maintain water homeostasis^48^,^49^. Proline is one of the micromolecular osmolytes and is an amino acid with the largest water solubility^48^. The proline content is very low under normal conditions, but high levels of proline content often accumulate in plants under abiotic stress^50^,^51^. Generally, proline content is considered to correlate to stress resistance of plants^52^. In this report, the proline contents of both TG and WT plants was higher when the salt concentration increased, but the proline content of WT plants increased more sharply, which indicated that the WT plants were more sensitive to salt stresses and needed to accumulate more proline to regulate homeostasis of water potential under the same salinity conditions^49^. In contrast, the TG plants were not as sensitive to salt stress and thus did not need to accumulate as much proline to resist salt stress. In addition, overexpressing *OsDST-SRDX* in transgenic plants may have mitigated the salt stress effects, and thus the TG plants were less stressed and therefore needed to accumulate less proline.

Plants have developed specific mechanisms such as ion-uptake regulation, vacuolar compartmentation and ion exclusion to survive when they encounter cellular ion imbalance caused by salt stress^53^. Salt tolerance was associated with a lower accumulation of Na^+^, and less Na^+^ accumulation has been used as a selective trait in breeding new varieties^54^,^45^. As reported in the rice *dst* mutant, Na^+^ uptake of the *dst* mutant was less than that of the WT plants and the plants showed significantly enhanced salt tolerance^26^. In this report, we observed that the *OsDST-SRDX* transgenic plants accumulated less Na^+^ than the WT plants when subjected to various concentrations of NaCl (100, 200 and 300 mM), indicating that the salt exclusion mechanism may play an important role in the salinity resistance of the transgenic plants. Also, less Na^+^ accumulation in the cytoplasm of the cell of transgenic plants led to less cell damage and might contribute to the enhanced salt tolerance of the *OsDST-SRDX* transgenic plants. Salinity damaged K^+^ homeostasis, as well. The *OsDST-SRDX* transgenic plants appeared to accumulate more K^+^ than the WT plants, indicating the *DST* transcription factor might affect genes related to ion transport. Despite that, the higher K^+^/Na^+^ ratio, which reportedly associated with salt tolerance, was also observed in the *OsDST-SRDX* transgenic plants.

Stomata were reported to make contributions to the responses of plants to various abiotic stresses^55^, and stomata can be induced to close by H_2_O_2_^56^. H_2_O_2_ is one of the key signaling molecules which participate in the complex signaling network and response to various abiotic stresses^56^. In our study, we examined the H_2_O_2_ content in the leaves of TG and WT plants before and after NaCl treatment by DAB staining and in both cases we found a higher accumulation of H_2_O_2_ in the TG plants. And after 4 days treatment of 200 mM of NaCl, the leaves of both the TG and the WT plants accumulated higher H_2_O_2_ content than the 0 mM NaCl condition (Fig. S4A). Furthermore, higher H_2_O_2_ levels in the leaves of the TG plants were observed under normal conditions according to an H_2_O_2_ kit (Fig. S4B). The higher H_2_O_2_ accumulation in the *OsDST-SRDX* transgenic plants might contribute to the salt tolerance of transgenic perennial ryegrass through a signaling network.

In summary, salt tolerance of transgenic perennial ryegrass was enhanced significantly through multiple physiological pathways by overexpressing a fusion gene, *OsDST-SRDX*. The transgenic perennial ryegrass could survive under 300 mM NaCl treatment for three weeks, which was associated with vigorous plant growth, higher leaf RWC, lower cell membrane leakage, lower MDA content, less Na^+^ accumulation and more K^+^ accumulation in the shoots. However, the molecular mechanisms of enhancing salt tolerance in perennial ryegrass by suppressing the function of the *DST* transcription factor remains to be elucidated.

## Materials and Methods

### Plant materials and transformation

Six DSTs derived from different plant species (*Hordeum vulgare*, *Panicum virgatum*, *Oryza sativa*, *Brachypodium distachyon*, *Setaria italic* and *Zea mays*) were used to construct a Neighbor-Joining phylogentic tree by using MEGA5.1. DNAMAN6.0 was used for the nucleotide sequence alignment of the *DST* genes of rice (*OsDST*), switchgrass (*PvDST*), brachypodium (*BdDST*), barley (*HvDST*), millet (*SiDST*), and maize (*ZmDST*). The conserved sequence of the alignment *DSTs* was used to design a pair of primers (LpDST F: 5′-AGAAGTTCCTCAAGTCGCAG-3′; LpDST R: 5′-TAGAAGTAGGGGTTCCAGCC-3′) to amplify the conserved sequence fragment of *DST* gene in perennial ryegrass (Citation IV). The cloned conserved sequence fragment of *LpDST* was aligned with the corresponding conserved domain of *OsDST*, *PvDST* and *BdDST* by DNAMAN6.0.

A semi-quantitative RT-PCR and a real-time quantitative PCR were applied with the pair of primers LpDST, cDNAs derived from the leaves of WT perennial ryegrass after 0 h, 0.5 h, 3 h, 6 h, 12 h and 24 h salinity treatment respectively under 300 mM NaCl as the template to evaluate the responses of the endogenous *DST* of perennial ryegrass to salt stress. The annealing temperature for RT-PCR was 54 °C; 28 reaction cycles were given. And a real-time quantitative PCR with the same primer and template was given to evaluate the relative expression level of *LpDST* gene under salt stress, the annealing temperature was 60 °C, and 40 reaction cycles were given.

Perennial ryegrass cultivar ‘Citation IV’ was used for transformation. Transgenic plants overexpressing the *OsDST-SRDX* fusion gene were produced via *Agrobacterium*-mediated transformation of embryonic callus derived from mature seeds of perennial ryegrass as described previously^15^. The *Agrobacterium* strain EHA105, harboring a binary vector pZH01_*OsDST-SRDX*, was used in the transformation. The selectable marker gene *hpt* and the *OsDST-SRDX* fusion gene in the T-DNA region were under the control of a CaMV 35S promoter, respectively. The SRDX domain (LDLDLELRLGFA) was linked to the C-terminal of *OsDST* gene after get rid of stop codon^30^,^31^. The calluses after infection were selected by 50 mg L^−1^ and 100 mg L^−1^ hygromicin B; resistant calluses were selected to transfer to a differentiation medium. The WT plants were also regenerated from tissue culture. Regenerated plantlets were transferred in pots filled with a mixture of soil and maintained in a growth-room under a 16/8 h (light/dark) photoperiod with 200 μmol m^−2^s^−2^ light intensity at 25 ± 2 °C.

### Molecular identification of transgenic plants

Transgenic perennial ryegrass plants were verified by PCR and RT-PCR assays. Total genomic DNA was extracted using the CTAB method^57^. Transgenic plants were identified by PCR with a pair of primers specific to the *hpt* gene (hpt-F: 5′-TACTTCTACACAAGCATCGGTCCAG-3′; hpt-R: 5′-CTTGACATTGGGGAGTTTAGCGAGA-3′) with a standard PCR program at an annealing temperature of 55 °C. Amplification products were separated on 1.0% (w/v) agarose gel. The positive transgenic plants were selected randomly for RT-PCR assays.

The total RNA was extracted using a plant RNA extraction kit (Takara Co. Dalian, China). One microgram of total RNA was treated with DNase I and used for cDNA synthesis using oligo (dT) primer and Superscript Reverse Transcriptase (Invitrogen). The cDNA was used as a template for semi-quantitative RT-PCR, which was performed with a pair of primers specific to the *OsDST* gene (OsDST-F: 5′-GGCTGTTCCCGTGCTTGTT-3′; OsDST-R: 5′-TCCTCGCCGTTGTTGCTG-3′). The annealing temperature for RT-PCR was 55 °C, and 30 reaction cycles were given. The amplified DNA products were separated on 2.0% (w/v) agarose gel. The perennial ryegrass internal reference gene was *LpActin*^58^.

### Phenotypic analysis of transgenic plants

After seven months when the WT and TG plants were all fully developed, four transgenic lines: TG4, TG5, TG6, TG7 and two wild-type lines, WT1 and WT2, were selected for comparison of plant height, leaf width and stem diameter. Fifteen individual plants generated from tillers of the same transgenic line were selected for each parameter measurement; 5 plants were put in one replicate, and three replicates were given in the experiments. The widest part of the mature leaf was measured as the leaf width; the stem diameter was measured at 3 cm above the ground.

### Salt-tolerance tests

The response of transgenic plants to salt shock was tested by directly watering them with 300 mM NaCl solution. The transgenic line TG7, showing obvious resistance to salt shock, was chosen for further detailed physiological detection to salt stresses. When transgenic plants grown in soil had enough tillers, the plants were removed from soil, and the soil was rinsed soil off the roots thoroughly, then consistent tillers were chosen and trimmed to a similar size and fixed into the holes of black-hole tray evenly, each hole being filled with 4 tillers. The hole-tray was put on the plastic containers (30 cm × 20 cm × 10 cm, packaged with black paper) containing 1/2× Hoagland nutrient solution, and the nutrient solution was just connected to the bottom of the tray. An air pump was used to aerate the nutrient solution continuously. The nutrient solution was changed once a week. The plants were maintained in the growth room under a 16 h light/8 h dark photoperiod with 200 μmol m^−2^s^−2^ light intensity at 25 ± 2 °C. Two weeks later, the roots of the plants were clipped to the same length (8 cm), and the plants above ground were trimmed to the same height also (15 cm) and placed in 1/2× Hoagland nutrient solutions supplemented with 0 mM, 100 mM, 200 mM, and 300 mM NaCl, respectively. Each gradient included 24 transgenic plants and 24 wild-type plants. Eight plants were treated as biological replicates. The variation of the morphology of the plants under salt stress conditions was observed and recorded every day. Physiological parameters, such as the vertical height of plants, the relative water content of leaves, electrolyte leakage, the proline content and the malondialdehyd (MDA) content were analyzed every four days.

### Measurement of relative leaf water content (RWC)

According to Li *et al.*^25^, leaves from TG and WT plants were harvested and weighed (*F*_*W*_) immediately. Then they were cut into pieces and immersed in Millipore water in a 10 mL tube at 4 °C for 24 h. After the turgid weight was measured (*T*_*W*_), the leaves were dried in an oven at 80 °C for 24 h and then weighed (*D*_*W*_) after cooling down. Finally, the RWC of leaves was estimated using the following formula: RWC = [(*F*_*W*_ − *D*_*W*_)/(*T*_*W*_ − *D*_*W*_)] × 100%.

### Measurement of electrolyte leakage (EL)

Electrolyte leakage was measured to evaluate the stability of the plant cell membrane. The conductance was used to estimate the amount of ions released from cells under various conditions. Following the description of Li *et al.*^25^, about 0.2 g of fresh leaves were harvested and cut into 1 cm segments. Then, after cleaning, they were immersed in 20 mL Millipore water in a 50 mL tube, and agitated for 24 h at room temperature. The electrical conductivity was measured by a conductometer (AZ pH/mV/Cond. /TDS/Temp. meter 86505). Then the mixture was autoclaved for 30 min for sterilization and agitated for 24 h at room temperature to measure the conductivity again. The electrolyte leakage (EL) = C_i_/C_max_ × 100%.

### Proline content measurement

Proline content was determined as described by Li *et al.*^25^ with minor modifications. Briefly, 0.2–0.5 g of fresh leaves were harvested, cut into pieces, placed in 5 mL 3% sulphosalicylic acid and held in boiling water for 10 min. Two mL extract was reacted with 2 mL glacial acid and 3 mL acid ninhydrin in a 20 mL glass tube for 45 min at 100 °C. The reaction mixture was extracted with 5 mL toluene and vortexed for 15 sec. The absorbance of the toluene layer was read at 520 nm in a Thermo Spectronic BioMate 3. Proline concentration was determined from a standard curve.

### MDA content measurement

About 0.5 g of fresh leaf fragments were harvested and ground thoroughly in 5 mL 10% TCA with a little quartz sand. The extraction was obtained after centrifugation at 3000 rpm for 10 min. Then 2 mL extract was reacted with 2 mL 0.6% barbituric acid at 100 °C for 15 min. An ice bath was used to terminate the reaction. The absorbance of the reaction mixture was read at 532 nm, 450 nm and 600 nm. MDA content was determined using the following formula^59^:


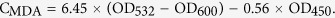


### Na^+^ and K^+^ content measurement

Shoot and root parts of perennial ryegrass under various salt concentrations (0, 100, 200 and 300 mM) were harvested respectively after 12 days of salt treatment and dried at 65 °C for 48 h. About 50 mg of dry powder samples were used for Na^+^, K^+^ content measurement. Eight mL of deionized water was added to the sample in a 10 mL plastic tube and held in boiling water for 30 min. The supernatant was transferred into a 50 mL plastic tube. Deionized water was added to the mixture, and it was boiled 3 times. Then, all of the supernatant was filtered and diluted with deionized water to 50 mL. After that, a flame spectrophotometer was used to measure the Na^+^ and K^+^ content^60^.

### Diaminobenzidine (DAB) staining and quantitative measurement of H_2_O_2_

H_2_O_2_ was detected by DAB staining intuitively as described previously^26^,^61^. The H_2_O_2_ contents quantitatively measured using a Hydrogen Peroxide assay kit (Njjcbio, A064-1) according to the manufacturer’s protocol.

### Statistical analysis

All data were subjected to one-way analysis of variance (ANOVA, SPSS 18.0), multiple comparisons of the mean value were made by Duncan.

## Additional Information

**How to cite this article**: Cen, H. *et al.* Overexpression of a Chimeric Gene, *OsDST-SRDX*, Improved Salt Tolerance of Perennial Ryegrass. *Sci. Rep.* **6**, 27320; doi: 10.1038/srep27320 (2016).

## Supplementary Material

Supplementary Information

## Figures and Tables

**Table 1:** Growth parameters of perennial ryegrass^1^.

	Plant height/cm	Stem diameter/mm	Leaf width/mm
WT1	9.667 ± 0.333c	1.975 ± 0.095b	2.203 ± 0.059d
WT2	11.867 ± 0.376c	2.238 ± 0.204b	2.740 ± 0.136c
TG4	20.420 ± 1.146ab	3.023 ± 0.261a	3.598 ± 0.073ab
TG5	20.600 ± 0.909ab	3.293 ± 0.323a	3.330 ± 0.061b
TG6	17.925 ± 0.767b	2.933 ± 0.073a	3.670 ± 0.121a
TG7	21.100 ± 0.682a	3.160 ± 0.169a	3.423 ± 0.085ab

^1^Each value represents the Mean ± SE from three replicates. The letters following the value in the same row indicate the differences between the TG and WT according to ANOVA analysis (*p* < 0.05).
