# Supplementary material for: Overexpression of a Chimeric Gene, OsDST-SRDX, Improved Salt Tolerance of Perennial Ryegrass
Source: Sci Rep. 2016 Jun 2;6:27320. doi: 10.1038/srep27320 (PMC4890315; doi:10.1038/srep27320)
Supplement: Supplementary Information [file srep27320-s1.doc]

**Supplementary Data**

**Overexpression of a** **Chimeric Gene, *OsDST-SRDX,* Improved Salt Tolerance of Perennial Ryegrass**

Huifang Cen^1**^, Wenxing Ye^1**^, Yanrong Liu^1^, Dayong Li^3^, Kexin Wang^1^, Wanjun Zhang^1, 2^*

*^1^ Beijing Key Laboratory for Grassland Science, Department of Grassland Science, China Agricultural University, Beijing, 100193,* *P.R China*

*^2^National Energy R&D Center for Biomass (NECB), China Agricultural University, Beijing, 100193, P.R China*

*^3^State Key Laboratory of Plant Genomics and National Center for Plant Gene Research, Institute of Genetics and Developmental Biology, Chinese Academy of Sciences, Beijing 100101, P.R China*

**Corresponding author, Email:* [*wjzhang@cau.edu.cn*](mailto:wjzhang@cau.edu.cn)*, Tel/Fax: 86 10 6273 3888.*

*** These authors contributed equally to this work.*

*
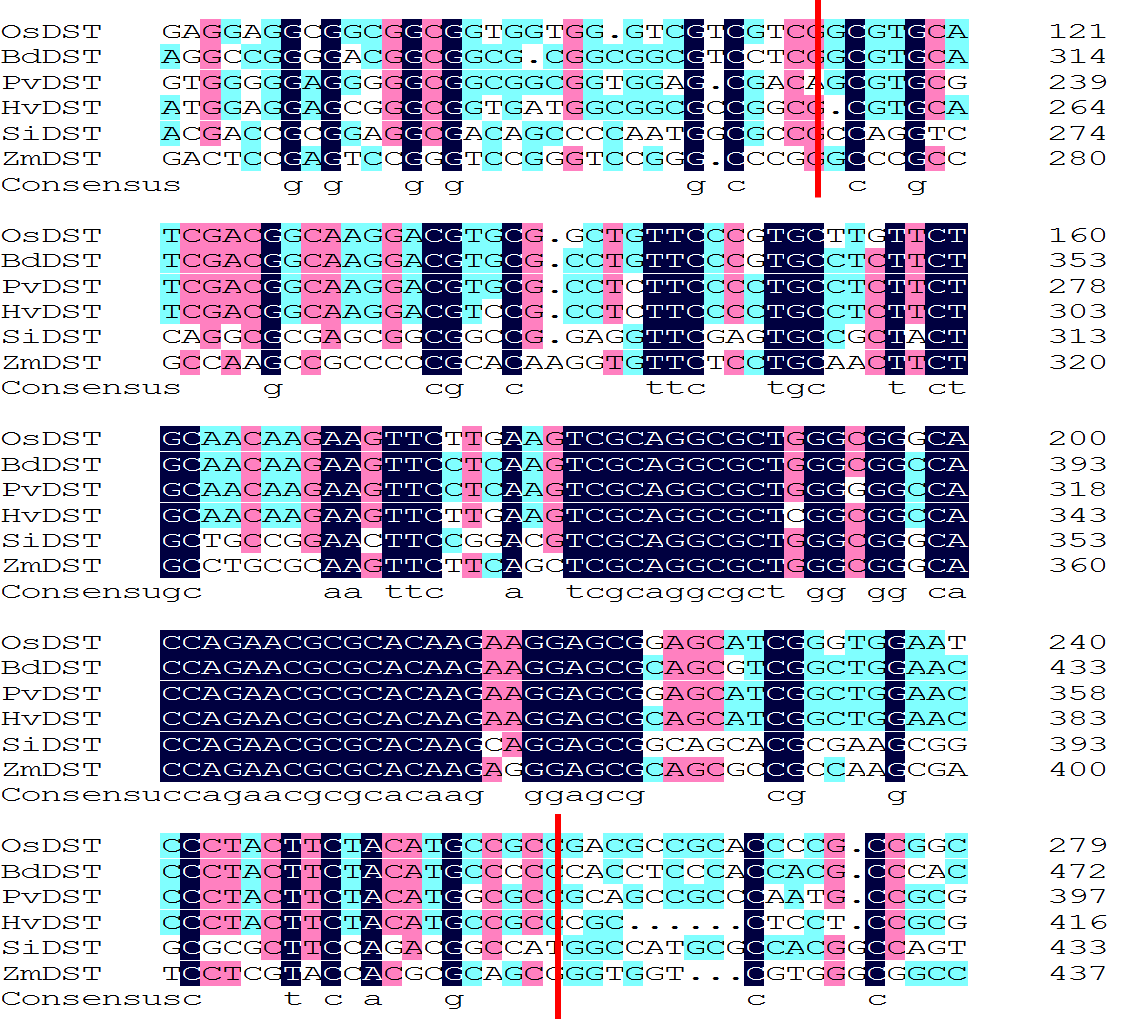
*

Figure S1 The nucleotide sequence alignment between *OsDST*, *PvDST*, *BdDST*, *HvDST, SiDST,* and *ZmDST.* The conserved sequence between the two red lines was used to design a pair of primers for LpDST amplification.


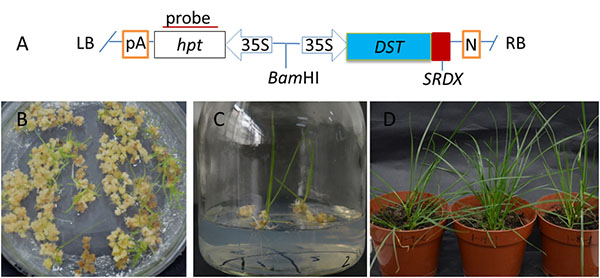


Figure S2 Generation of the transgenic perennial ryegrass

A, schematic diagram of the T-DNA region of chimeric gene pZH01_*OsDST-SRDX*; B, somatic-embryos induction; C, resistant perennial ryegrass plantlets rooting on MS medium supplied with 50mg L^-1^ *hpt*; D, resistant regeneration plantlets in pots.


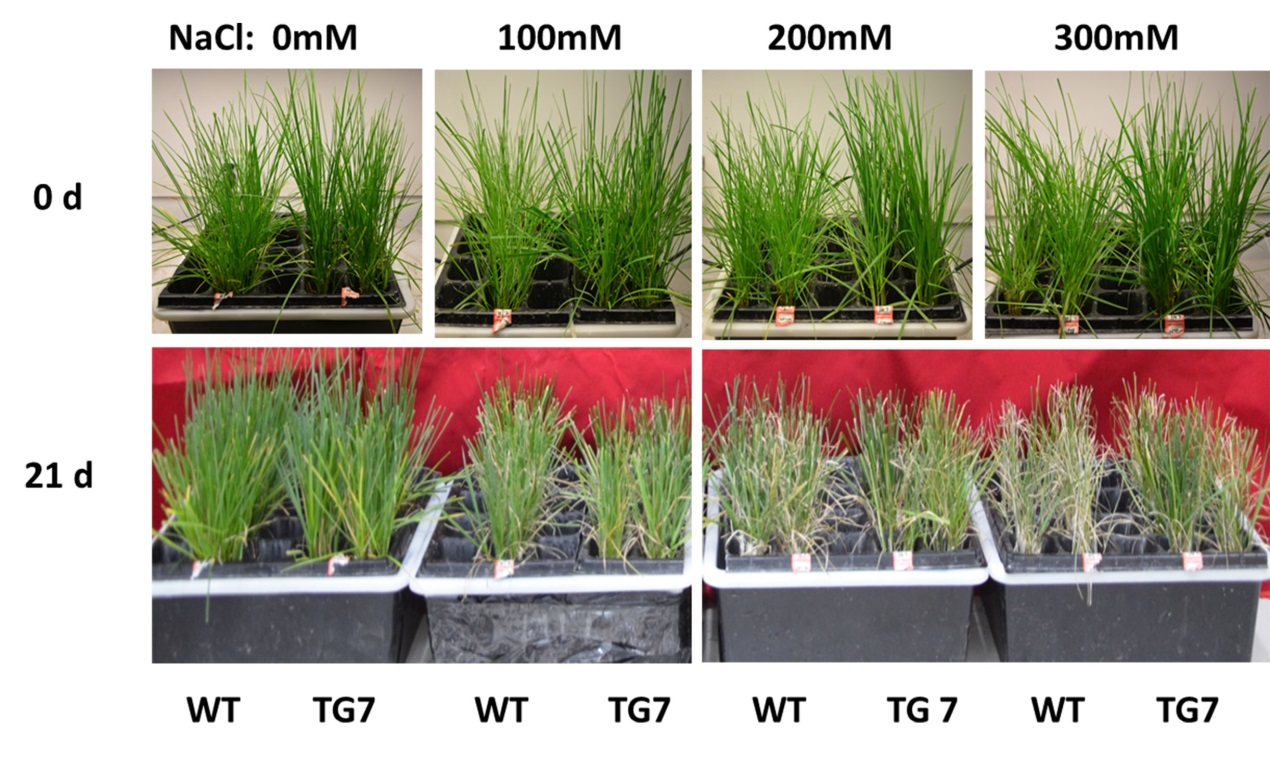


Figure S3 Phenotype of TG and WT plants under various concentration of NaCl treatment before and after 21 days


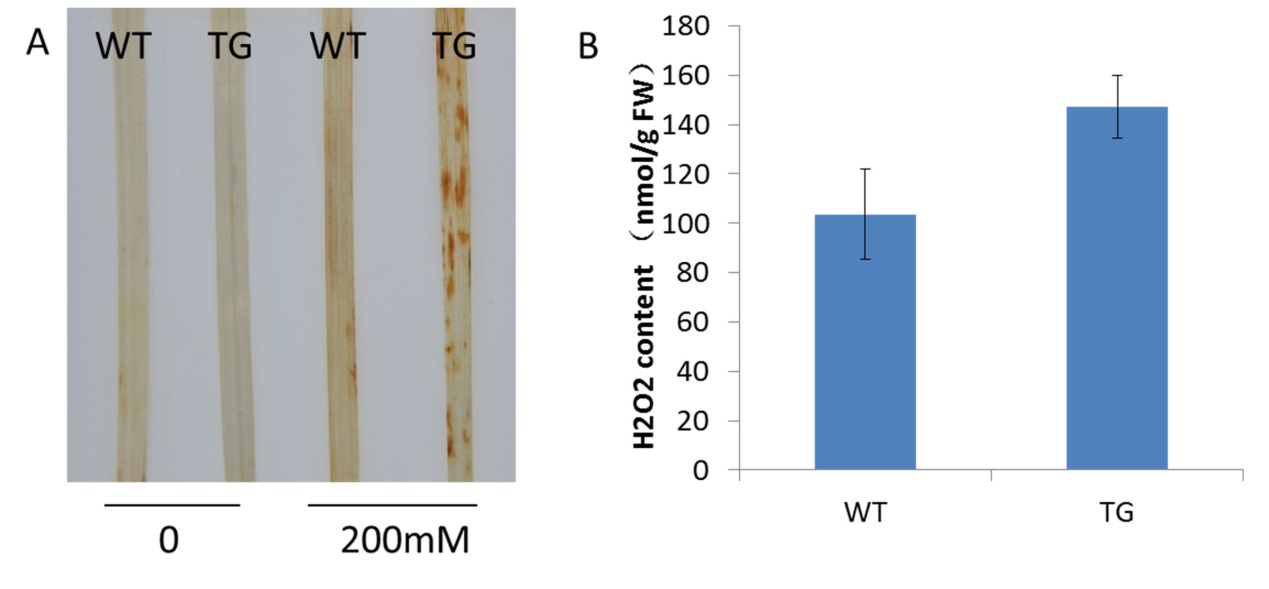


Figure S4 Measurement of H_2_O_2_ content of TG and WT plants

A, DAB staining of mature leaves of TG and WT plants under normal and 200 mM salinity conditions; B, quantitative measurement of H_2_O_2_ in the mature leaves of WT and TG plants by using a H_2_O_2_ kit
